# Li_2_O-Reinforced Solid Electrolyte Interphase on Three-Dimensional Sponges for Dendrite-Free Lithium Deposition

**DOI:** 10.3389/fchem.2018.00517

**Published:** 2018-11-06

**Authors:** Chao Shen, Huibo Yan, Jinlei Gu, Yuliang Gao, Jingjing Yang, Keyu Xie

**Affiliations:** ^1^State Key Laboratory of Solidification Processing, Center for Nano Energy Materials, School of Materials Science and Engineering, Northwestern Polytechnical University and Shaanxi Joint Laboratory of Graphene (NPU), Xi'an, China; ^2^School of Materials and Chemical Engineering, Xi'an Technological University, Xi'an, China

**Keywords:** CuO@Cu, submicron wire sponge, current collector, lithium anode, dendrite-free

## Abstract

Lithium (Li) metal, with ultra-high theoretical capacity and low electrochemical potential, is the ultimate anode for next-generation Li metal batteries. However, the undesirable Li dendrite growth usually results in severe safety hazards and low Coulombic efficiency. In this work, we design a three-dimensional CuO@Cu submicron wire sponge current collector with high mechanical strength SEI layer dominated by Li_2_O during electrochemical reaction process. The 3D CuO@Cu current collector realizes an enhanced CE of above 91% for an ultrahigh current of 10 mA cm^−2^ after 100 cycles, and yields decent cycle stability at 5 C for the full cell. The exceptional performances of CuO@Cu submicron wire sponge current collector hold promise for further development of the next-generation metal-based batteries.

## Introduction

Research has been focused on practical applications of Li metal anode since 1970s, due to its theoretical capacity (3,860 mAh g^−1^) and low electrochemical potential (−3.040 V vs. SHE; Li et al., 2014; Mukherjee et al., 2014; Cheng et al., 2017; Shen et al., 2018). Unfortunately, the commercialization of Li metal anode has been retarded for several decades by the problem of Li dendrites which cause poor Coulombic efficiency and mass capacity loss (Amine et al., 2014). More seriously, the sharp Li dendrites will pierce through the separator, generating internal short-circuit, bringing about severe safety hazards (Bouchet, 2014; Lu et al., 2014). It is well accepted that the growth of Li dendrites is mainly attributable to two reasons (Bouchet, 2014; Xu et al., 2014; Aryanfar et al., 2015; Cheng et al., 2017). (1) The solid electrolyte interphase (SEI) layer forms on the Li metal anode with insufficient mechanical strength. Li reacts instantly in contact with liquid electrolytes and rapidly forms an SEI film. This passivation layer of SEI prevents further loss of Li and electrolyte caused by their continued reaction (Lu et al., 2015; Shin et al., 2015). However, natural SEI layer does not have enough mechanical strength to withstand large volume change during Li charge/discharge processes (Liang et al., 2015). (2) Inhomogeneous distribution of Li^+^ on anode. Li^+^ accumulates and deposits on the “hot spots” because of the roughness of current collector, eventually forms numerous Li dendrites (Lin et al., 2016; Shen et al., 2018).

Scientists proceed from above two reasons to address the Li dendrites problems, and have achieved solid progress on suppressing dendrite formation and growth (Camacho-Forero et al., 2015; Lu et al., 2015; Shin et al., 2015; Zu et al., 2016; Jin et al., 2017; Liu et al., 2017). Cui summarized traits indispensable for an ideal SEI layer (Liu et al., 2017a). (1) Homogeneity in all aspects. (2) High modulus and compact structure. (3) Flexibility to accommodate the ineluctable interface fluctuation during battery cycling. (4) High ionic conductivity. Natural SEI can hardly meet all the requisites above, hence necessitates the rational design of SEI. Various electrolyte additives and artificial SEI films have been employed to reinforce the SEI layer and suppress the formation of Li dendrites. Additives currently proven effectual mainly include vinylene carbonate (Ota et al., 2004; Stark et al., 2011), fluoroethylene carbonate (Liu et al., 2015), LiNO_3_ and lithium polysulfide (Li et al., 2015), lithium fluoride (Choudhury and Archer, 2016), ionic liquid (Schweikert et al., 2013), metal ions (Ding et al., 2013), as well as trace amount of water and gases (Christensen et al., 2012; Qian et al., 2015). The concept of artificial protective film has been deeply explored in previous studies, and various artificial films have been applied on Li foil surfaces, such as lithium polyacetylene (Sakamoto et al., 2001), tetraethoxysilane (Umeda et al., 2011), lithium phosphorus oxynitride (Dudney, 2000), Cu_3_N nanoparticles compounded styrene butadiene rubber (Liu et al., 2017a), a hollow carbon nanospheres layer (Zheng et al., 2014), a boron nitride layer (Luo et al., 2015), a modified poly (dimethylsiloxane) film (Zhu et al., 2017), and a Li_3_PO_4_ layer (Li et al., 2016). However, stable cycling cannot be guaranteed due to the consumption of additives in long-term cycle, and the artificial protective film can increase the impedance and reduce specific energy density.

Therefore, it is necessary to obtain a SEI layer with high mechanical strength to suppress Li dendrites effectively. In general, the SEI films dominated by inorganic crystalline components such as Li_2_CO_3_ (Fujieda et al., 1994; Shang et al., 2012), Li_2_O (Billone et al., 1986; Zhang et al., 2016), and LiF (Combes et al., 1951; Takehara, 1997) are strong in mechanical strength while those dominated by organic components such as lithium alkyl carbonates (ROCO_2_Li) are found to be porous and fragile with low shear modulus under 1 GPa (Stone et al., 2012; Karkera and Prakash, 2018). Theoretical predictions have shown that a solid film with elasticity modulus of 1 GPa should be sufficient in suppressing dendrites (Monroe and Newman, 2005; Stone et al., 2012). M.C. Billone etc. reported that Li_2_O has a high elasticity modulus of 108 GPa (Billone et al., 1986), much higher than the threshold. Furthermore, Zen-ichiro Takehara proved that the Li_2_O containing SEI layer is adjacent to the Li metal anode (Park et al., 2017).

However, a dentrite-free morphology requires not only a reinforcement SEI but also homogenized electric field which is crucial for the uniform deposition of Li. Other approaches exploring 3D conductive carbon-based and metal-based current collectors to achieve uniform Li^+^ deposition and adapt to volumetric change during Li plating/stripping. Scientists have gained ground on the conversion of Cu foil into a 3D host current collector such as 3D porous Cu (Yun et al., 2016), Cu nanoclusters structure (Zhang et al., 2016) and aligned CuO nanosheets on a planar Cu foil (Zhang et al., 2018). The carbon-based 3D current collectors include nitrogen-doped graphene (Zhang et al., 2017), nanoparticles anchored on carbon nanofibers (Yang et al., 2017), and hollow carbon spheres (Shen et al., 2018). Recently, Wei et al. found that the tortuous pores of the porous media can drastically reduce the local flux of Li^+^ moving toward the anode and effectively extend the physical path of dendrite growth (Li et al., 2018). These studies reveal that 3D current collectors and inter-layer can homogenize the Li metal deposition, therefore suppress the formation of Li dendrites.

Herein, we design a three-dimensional (3D) porous CuO@Cu submicron wire sponge to inhibit the formation of Li dendrites. The 3D CuO@Cu submicron wire sponges own unique porous microstructure, which can homogenize the distribution of charges and inhibit the dendrite growth. Furthermore, Li_2_O gradually forms adjacent to the surface of Cu during the electrochemical reaction process of 2Li+CuO → Li_2_O+Cu. As a result, the 3D CuO@Cu collector within a SEI film dominated by Li_2_O is not only helpful for the enhancement of Li^+^ diffusion kinetics, but also beneficial for suppressing the Li dendrite due to the high shear modulus and rather strong mechanical strength.

## Materials and methods

### Synthesis of the porous 3D CuO@Cu submicron wire sponges

The precursor materials were composed of sodium hydroxide (NaOH, 40 mL 15 M), copper sulfate (CuSO_4_, 200 μL 1 M), ethylenediamine (EDA, 300 μL 99 wt%), and hydrazine (50 μL 35 wt%), and the precursor suspension was initially dispersed by ultrasonication and added to a plastic vessel. The sealed plastic vessel was heated at 70°C in a water bath for 12 h to form a continuous Cu submicron wire hydrogel. After that, the as-synthesized Cu submicron wire hydrogel was washed with hydrazine solution (5wt%) several times to remove NaOH. Then, the as-synthesized Cu submicron wire hydrogel was treated for 5 min in constant temperature and humidity test chamber (60°C, 80%). Finally, the CuO@Cu hydrogel was frozen and dried into sponges to retain the original gel volume.

### Characterizations

Field emission scanning electron microscopy (FESEM) measurements were carried out with Nova NanoSEM 450 equipped with an EDX spectroscopy attachment. X-ray diffraction (XRD) was recorded from 20 to 85° on a Bruker D8 advance diffractometer with CuKα radiation (λ = 1.5406 Å). The contact angle was measured by an Optical Contact Angle & interface tension meter (SL200KB, Kino, USA) at room temperature in air, and a 3.0 μL droplet of the ether-based electrolyte was used in the experiment.

### Electrochemical measurements

The 3D porous CuO@Cu submicron wire sponge was first pressed and punched out into circular disks with a diameter of 12 mm as 3D porous current collectors for Li metal anodes. For repeated Li deposition/stripping testing, CR2032 coin cells were assembled using a 3D porous CuO@Cu submicron wire sponge or a planar Cu foil as the working electrode, a Li foil as the counter electrode, and a Celgard microporous polypropylene film as the separator. The Li deposition capacity is fixed at 1.0 mAh cm^−2^ and the cut-off potential for the stripping process is configured to be 1.0 V. The electrolyte was 1 M lithium bis(trifluoromethane sulfonyl)imide (LiTFSI) in cosolvent of 1,3-dioxolane (DOL) and 1,2-dimethoxyethane (DME; 1:1 in volume) with 2% LiNO_3_. For the symmetrical cell test, 1 mAh cm^−2^ of Li was first plated onto the current collectors at a current density of 2 mA cm^−2^, then the cells were cycled at a current density of 0.5 mA cm^−2^ for 0.5 h in each half cycle. For the LiFePO_4_ full cells, the LiFePO_4_ electrodes were prepared by mixing LiFePO_4_, polyvinylidene fluoride, and carbon black in the ratio of 8:1:1 with N-methyl-2-pyrrolidone as the solvent. The areal mass loading of the LiFePO_4_ electrodes was about 4.2 mg cm^−2^. The electrolyte is consisted of 1.0 M LiPF_6_ in ethylene carbonate (EC)/dimethyl carbonate (DMC) (1:1 in volume). The 3D porous CuO@Cu submicron wire sponges or planar Cu foil as a current collector was first assembled into a half cell using a Li foil as the counter electrode. After depositing 5 mAh cm^−2^ of Li metal onto the current collector, the cell was disassembled in an Ar-filled glove box, then the deposited Li current collector as anode was further reassembled into a full cell against LiFePO_4_ cathode. The LiFePO_4_ full cells were galvanostatically cycled between 2.4 and 4.3 V at 1 C. All the cells were tested using a CT2001A cell test instrument (LAND Electronic Co, BT2013A, China) or an 88-channel battery tester (Arbin Instruments, BT2000, USA). The cyclic voltammetry (CV) curves were measured with Solartron. For the Li||3D CuO@Cu CV curves, the voltage sweep rate was 0.1 mV s^−1^ between 0.01 and 3 V vs. Li/Li^+^. For full-cell CV curves, the voltage sweep rate was 0.1 mV s^−1^ between 2.4 and 4.3 V vs. Li/Li^+^.

## Results and discussion

**Figure 1:**
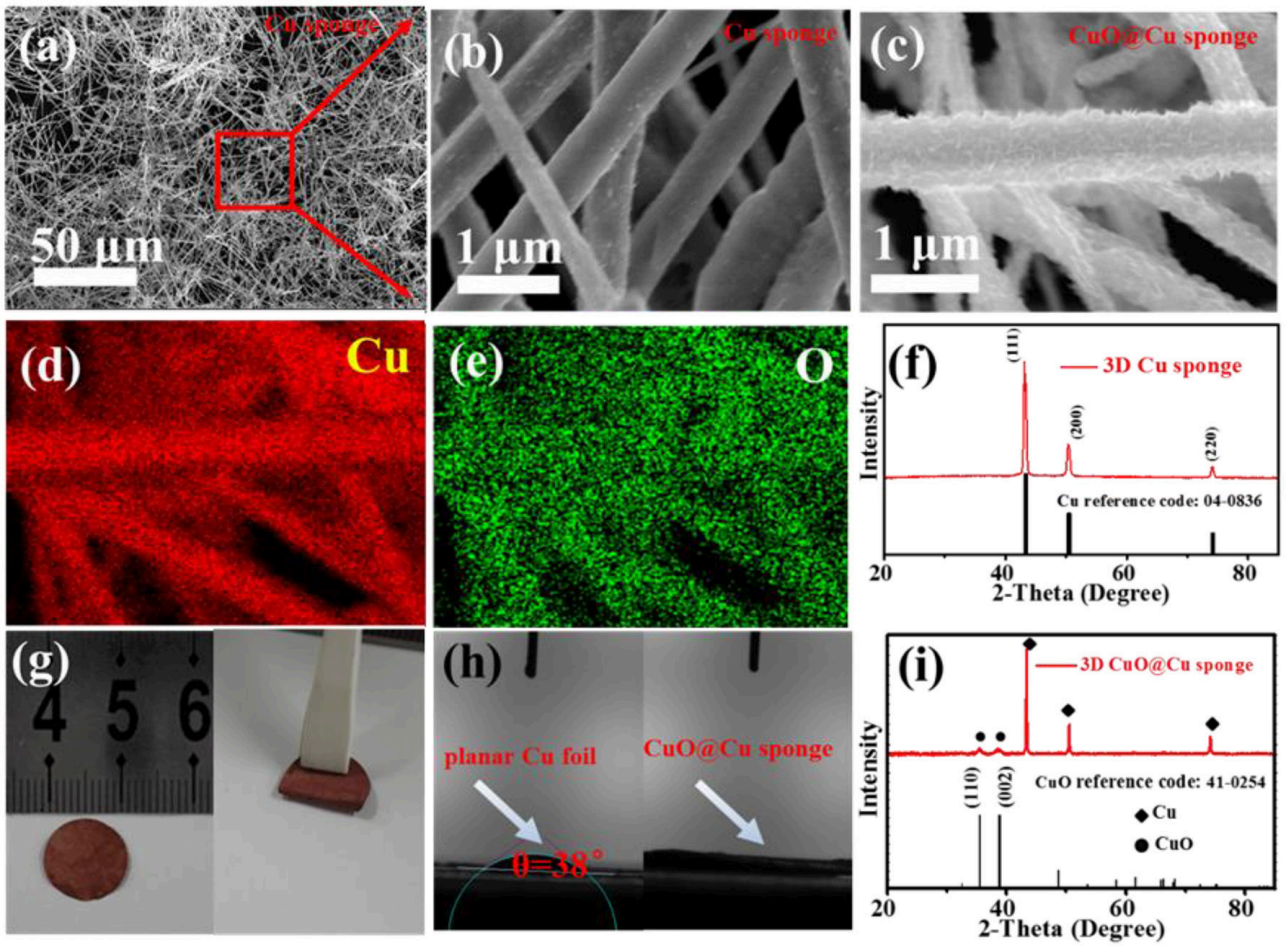
**(a)** Low- and **(b)** high-magnification FESEM images of the 3D porous Cu submicron wire sponge, respectively. **(c)** FESEM images of the 3D porous CuO@Cu submicron wire sponge. **(d,e)** Corresponding elemental mapping of Cu and O in **(c)**. **(f)** XRD pattern of the as-synthesized 3D porous Cu sponge. **(g)** Digital photos of 3D porous CuO@Cu submicron wire sponge after compressed (left), and folded (right). **(h)** Contact angles of LiTFSI-based electrolyte on the planar Cu foil and CuO@Cu submicron wire sponge. **(i)** XRD pattern of the as-synthesized 3D CuO@Cu submicron wire sponge.

The morphologies of the 3D porous Cu submicron wire sponge are shown in Figures 1a,b. It can be seen that as-synthesized Cu sponge consists of plenty of long intertwined submicron wires (Figure 1a). These wires, with length more than 100μm, have an average diameter of about 450 nm (Figure 1b) and the surface is very smooth. As shown in Figure 1f, the XRD was then employed to study the phase structure of the Cu submicron wires sponge. The patterns for Cu wires sponge is consistent with the JCPDS date (PDF#04-0836), indicating that Cu sponges have a face-centered cubic structure (Gao et al., 2001; Yu et al., 2005). In addition, there are three diffraction peaks at around 43.2°, 50.5°, and 74.1° correspond to the (111), (200), and (220) planes of the copper. Different from surface topography of 3D Cu wires, the high-magnification FESEM of the as-synthesized CuO@Cu submicron wire sponge shows that the fiber surface is rough (Figure 1c), and the corresponding elemental mapping further confirms the distribution of Cu and O (Figures 1d,e). As shown in Figure 1i, the XRD of CuO@Cu has two more obvious different diffraction peaks at around 35.5°and 38.9° compare with Cu submicron wire sponge, which are highly consistent with the (110) and (002) lattice plane attribute to CuO (PDF#41-0254), confirming the presence of CuO on the surface of Cu submicron wire (Liu et al., 2006; Yu et al., 2013; Zhang et al., 2016). As shown in Figure 1g, the 3D porous CuO@Cu submicron wire sponge exhibits decent mechanical and processing properties, it can be easily folded and blended without fracture. To evaluate the wettability between the electrolyte and 3D porous CuO@Cu submicron wire sponge, the contact angles of LiTFSI-based electrolyte on the planar Cu foil and 3D porous CuO@Cu submicron wire sponge were measured (Figure 1h). The contact angle of electrolyte droplet on the planar Cu foil is 38° (Figure 1h, left), while it is nearly 0° on the 3D porous CuO@Cu submicron wire sponge (Figure 1h, right), indicating a better wettability between the CuO@Cu submicron wire sponge and the electrolyte.

**Figure 2:**
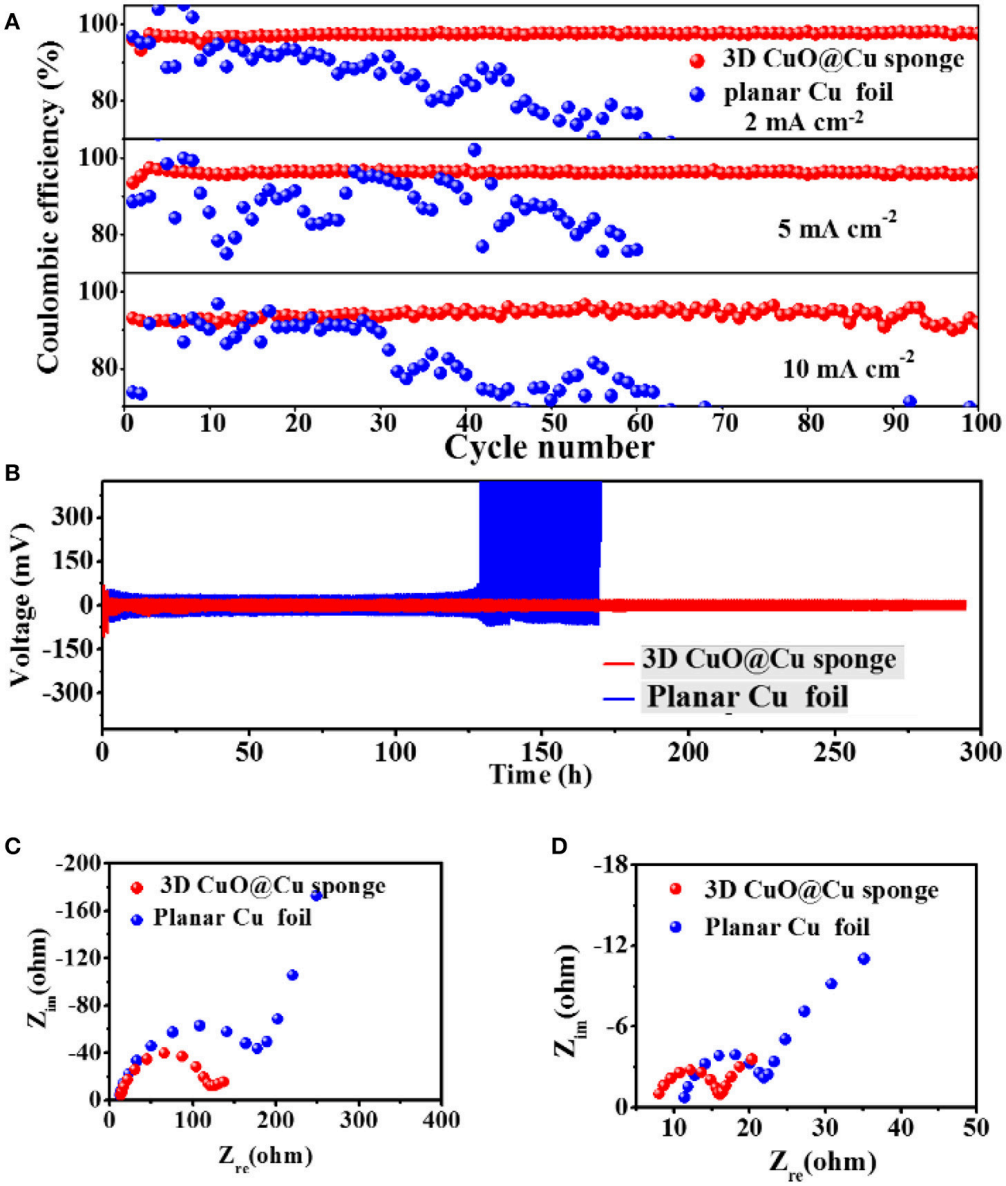
**(A)** Coulombic efficiencies of Li deposition/stripping on planar Cu foil and 3D CuO@Cu sponge current collectors at current density of 2, 5, and 10 mA cm^−2^, respectively. The deposition capacity of Li is fixed at 1 mAh cm^−2^. **(B)** Voltage profiles of Li metal deposition/stripping at current density of 0.5 mA cm^−2^ in symmetric cells with the planar Cu foils or the 3D CuO@Cu sponge as the current collectors. **(C,D)** The EIS curves of after initialization process and after 100 cycles at current density of 5 mA cm^−2^.

The electrochemical performances of the cells, with planar Cu foil and 3D porous CuO@Cu submicron wire sponge as current collectors, confirm that 3D porous CuO@Cu submicron wire sponge can effectively inhibit Li dendrites and exhibits better electrochemical performance. All the cells were first cycled from 0 to 1 V at 50 μA to remove surface contamination and stabilize the SEI film (Xu et al., 2014; Li et al., 2016). Coulombic efficiencies are shown in Figure 2A. At current density of 2, 5, and 10 mA cm^−2^, the Coulombic efficiencies of the planar Cu foil remain in a relative stable level (80–90%) within 30 cycles, and gradually decreased or fluctuated in the subsequent cycles, as a result of the SEI films are sabotaged by Li dendrites (Gao et al., 2001; Yu et al., 2005). In contrast, the Coulombic efficiencies of the 3D porous CuO@Cu submicron wire sponge current collector remain as high as 98% at current density of 2 mA cm^−2^, and 96% at current density of 5 mA cm^−2^ after 100 cycles. Even at an ultrahigh current density of 10 mA cm^−2^, the Coulombic efficiency still remains at 91% after 100 cycles. Cycling stabilities have been further investigated by symmetric cell test, which is a common technique to evaluate the characteristics of the interface in electrochemical devices (Zhu et al., 2017). The voltage profiles of metallic Li deposition/stripping in symmetric cells with planar Cu foil or 3D porous CuO@Cu submicron wire sponge are shown in Figure 2B, and the 3D CuO@Cu submicron wire sponge shows much more stable cycling than its planar Cu foil counterpart with severe fluctuations due to the polarization caused by the repeatedly breaking and repairing the SEI film (Monroe and Newman, 2005). In other words, a stable SEI film, as well as long-term cycling stability, can be realized in the symmetric cell test with 3D porous CuO@Cu submicron wire sponge. The prominent electrochemical properties of 3D CuO@Cu submicron wire sponge can be further confirmed by the electrochemical impedance spectroscopy (EIS) analysis conducted on after initialization process (Figure 2C) and the 100th cycles at current density of 5 mA cm^−2^ (Figure 2D). The diameter of semicircle at high frequency range is an indicator of the SEI film resistance. The SEI film resistance of the 3D Cu submicron wire sponge current collector is always lower than that of the planar current collector, indicating that the porous structure of the 3D current collector is beneficial for the kinetics of electrochemical reactions of electrodes (Monroe and Newman, 2005). It is worth noting that, after initialization process and 100th cycles of Li deposition/dissolution, the SEI film resistance reduces. The drop in resistance is associated with residual lithium on the current collector that increase the interfacial area between the electrolyte and lithium metal, which results in the reduction of resistance of Li/electrolyte interface.

**Figure 3:**
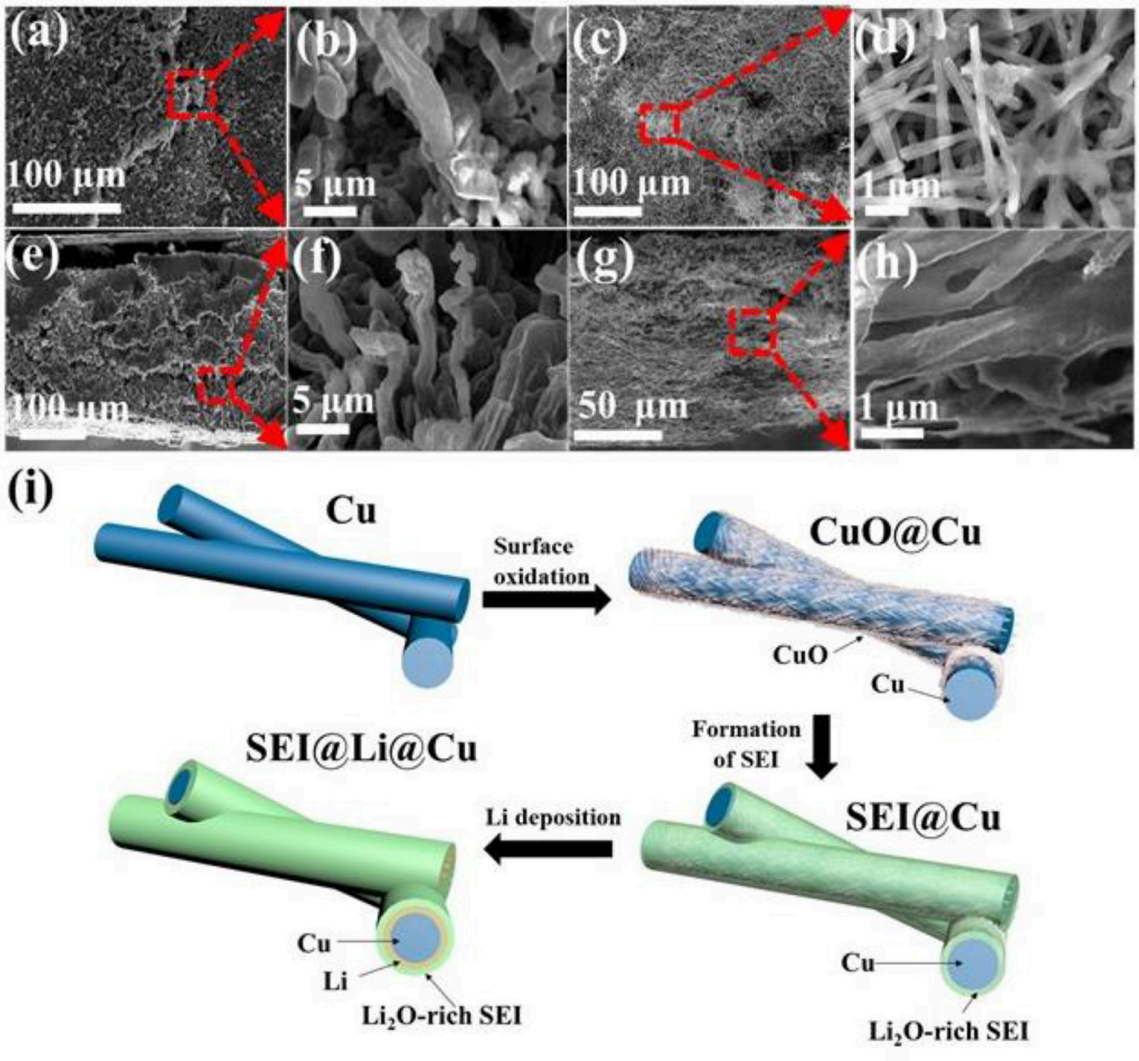
FESEM images of Li deposited onto planar Cu foil and 3D porous CuO@Cu submicron wire sponge current collectors after 100 cycles at current density of 5 mAh cm^−2^. **(a)** Top-view and **(e)** cross-sectional FESEM images of Li deposits on the planar Cu foil. **(b,f)** Magnified views of Li deposits in **(a,e)**, respectively. **(c,g)** Top-view and cross-sectional FESEM images of Li deposits within the 3D porous CuO@Cu submicron wire sponge. **(d,h)** Magnified views of Li deposits in **(c,g)**, respectively. **(i)** Schematic illustration of Cu submicron wire sponge is treated in constant temperature and humidity test chamber to obtain CuO@Cu submicron wire sponge. A Li_2_O-rich SEI layer is formed when Li^+^ encounters CuO during the electrochemical reaction process. Li^+^ passes through the Li_2_O-rich SEI layer and adheres to the Cu submicron surface and acquires an electron and becomes Li^0^.

As shown in Figure 3, after 100 cycles, the morphologies of Li deposition on planar Cu foil current collector and 3D porous CuO@Cu submicron wire sponge current collector are totally different. There are a great number of fiber-like Li dendrites with length of more than 10 μm and width of 3 μm on the surface of planar Cu foil current collector (Figures 3a,b). These Li dendrites could short-circuit the cell and cause safety hazard. It can be observed from the cross-sectional FESEM images that the original compact Li metal has become porous after cycles (Figures 3b,f), some of them may become electrically isolated and eventually form so-called “dead” Li, resulting in low Coulombic efficiency and rapid capacity loss (Zhang et al., 2017). However, compared to planar Cu foil covered with lots of mossy and dendritic Li after repeated deposition and stripping cycles, Li deposition is compact on the surface of CuO@Cu submicron wires (Figures 3d,h). According to the Figure 3e and Figure S1A, the Li adhered on the current collector is 200 μm thick after 100 cycles. In other words, the thickness of the Li-planar Cu foil anode is increased by 200 μm. However, the thickness of 3D porous CuO@Cu current collector hardly changes after 100 cycles (Figure 3g and Figure S1B). Thus, the 3D porous structure CuO@Cu current collector can adapt to volume changes during charging and discharging process. The submicron wire structure could homogenize the electric field distribution, as a result, uniform Li deposition is expected to cover the submicron wire surface. Meanwhile, Li_2_O enhances the mechanical strength of the SEI layer and is helpful for the enhancement of Li^+^ diffusion kinetics. During the charge/discharge process, Li^+^ gains electrons and eventually deposits between the SEI layer and the Cu surface. As a result, formation of Li dendrites is inhibited effectively.

**Figure 4:**
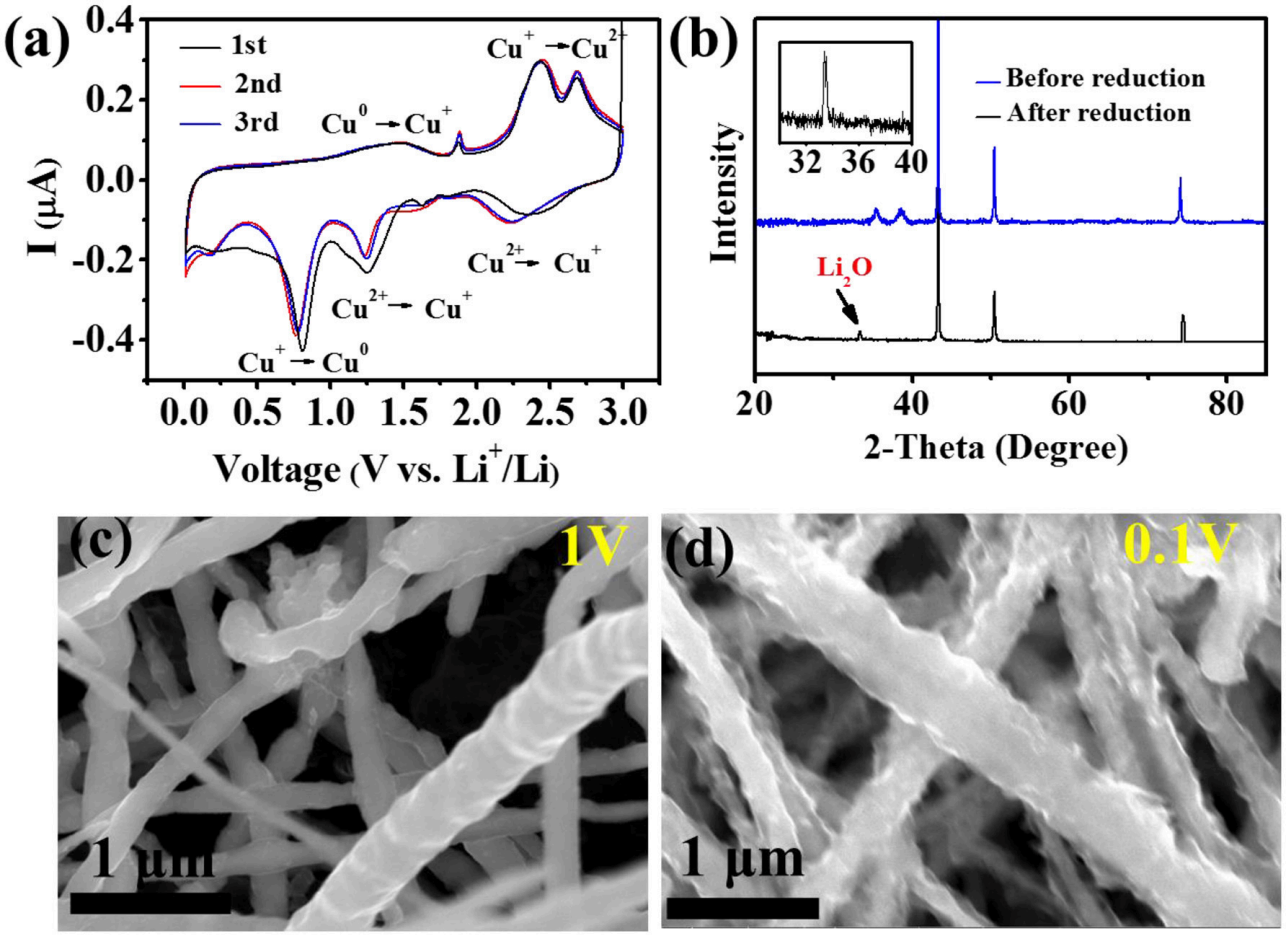
**(a)** The CV curves of Li||3D porous CuO@Cu sponge. **(b)** XRD of the CuO@Cu electrode before and after the electrochemical reduction. **(c,d)** FESEM images of the 3D porous CuO@Cu submicron wire sponge during the first reduction sweep at 1V and 0.1V, respectively.

Reduction sweep CV curves in Figure 4a corresponding to the conversion of CuO + 2Li^+^ + 2e^−^ → Cu + Li_2_O, which is identified by the redox peaks in CV (Gao et al., 2004; Zhang et al., 2016). During the first reduciton process, all Cu^2+^ is reduced to Cu^+^ at 1 V and all Cu^+^ is reduced to CuO at 0.1 V. In order to confirm whether or not CuO was transformed, we tested the XRD of the CuO@Cu submicron wire sponge before and after reduction. Figure 4b presents the XRD of the electrode before and after the electrochemical reduction of the CuO. The disappearance of the CuO phase justifies the reduction of CuO. The weak signal of Li_2_O after the reduction should be attributed to the formation of Li_2_O during reduciton process. FESEM images of CuO@Cu submicron wire sponge in Figures 4c,d indicate that CuO on the rough CuO@Cu surface is reduced to Cu and Li_2_O, gradually forming a smooth layer of SEI@Cu at 1 V. When the potential drops to 0.1 V, a thick Li_2_O-reinforced SEI can be clearly seen due to a serious amount of Li_2_O deposition. Wang reported that *in-situ* filled with Li_2_O on SEI layer formed by the CuO + 2Li^+^ + 2e^−^ → Cu + Li_2_O chemical reaction has a rather strong mechanical strength, which dendrite Li can hardly penetrate (Zhang et al., 2016). Guruprakash Karkera reported an *in situ* formed shielding layer composed of Li_2_O by 2Li+ + 2e^−^ + ½ O_2_ → Li_2_O which keeps the Li anode intact during vigorous cell conditions, providing faster Li-ion diffusion kinetics and stable cycling performance (Karkera and Prakash, 2018). Therefore, we propose that Li^+^ passes through the Li_2_O layer and adhere to the Cu surface, forming a Li layer (Figure 3i).

**Figure 5:**
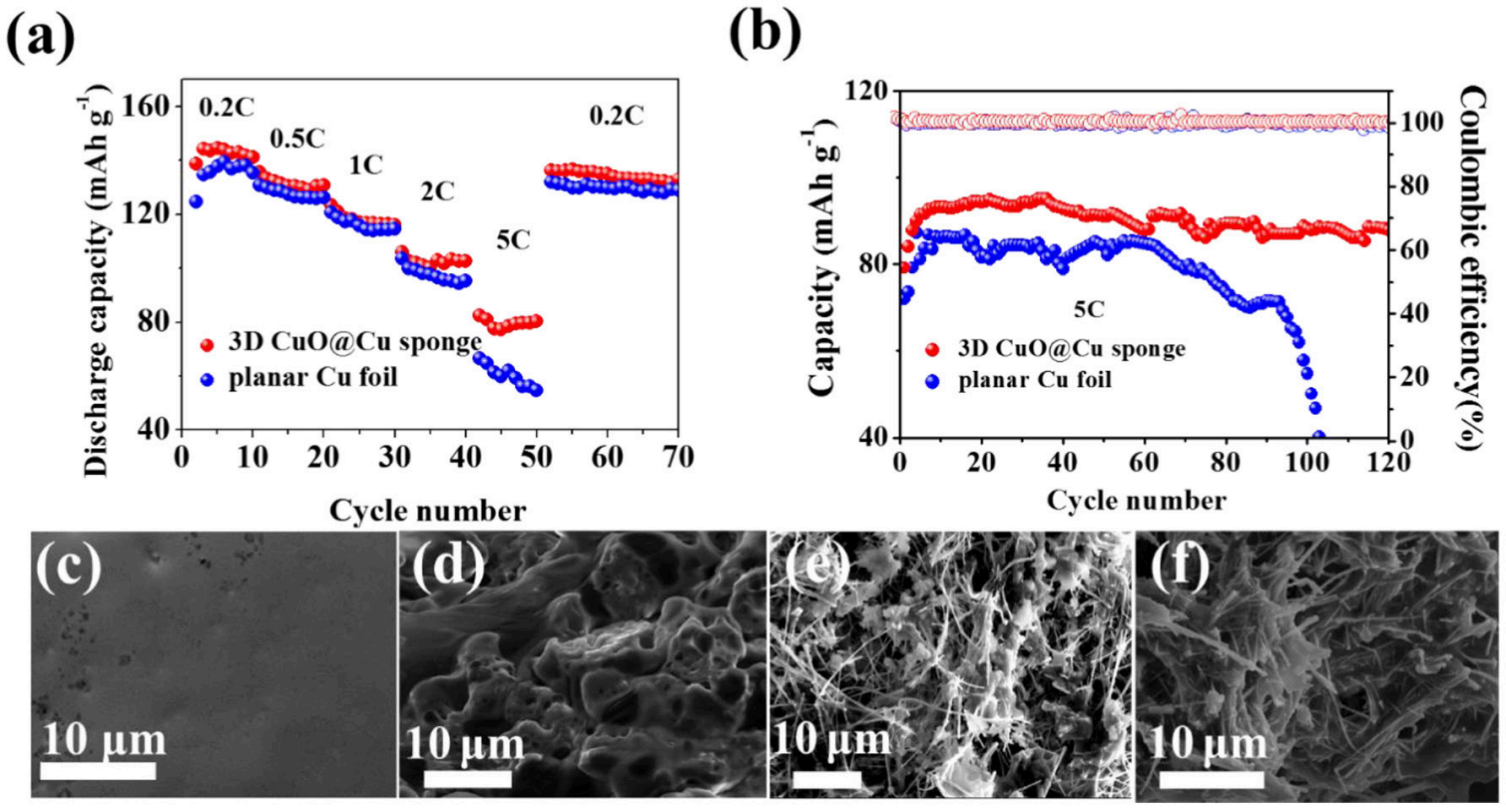
**(a)** Rate capabilities and **(b)** cycling performances of the full cells with a LiFePO_4_ cathode and different anodes (Li-planar Cu foil and Li-3D CuO@Cu sponge). **(c,d)** SEM images of the Li-planar Cu foil anode before and after cycling. **(e,f)** SEM images of the Li-3D CuO@Cu sponge anode before and after cycling.

To demonstrate the potential practical application of the 3D porous CuO@Cu submicron wire sponge current collector, full cells were built with the LiFePO_4_ as cathodes and Li-3D CuO@Cu sponge as anodes. The CV curves of Li-planar Cu foil || LiFePO_4_ and Li-3D CuO@Cu sponge || LiFePO_4_ are shown in Figure S2. It can be seen that there is only one peak pair, consisting of one anodic peak and one cathodic peak, which corresponds to the two-phase charge/discharge reaction of the Fe^3+^/Fe^2+^ (Yuan et al., 2011). Except for the first cycle, the CV curves of the cyles are almost coincident, indicating the preferable stability of the Li-3D CuO@Cu sponge and Li-planar Cu foil electrodes. For Li-3D CuO@Cu sponge anode, the voltage separation gets smaller than Li-planar Cu foil anode, indicating 3D CuO@Cu sponge anode is helpful for the enhancement Li^+^ diffusion kinetics and a low voltage hysteresis (Liu et al., 2017b; Su et al., 2018). As displayed in Figure 5a, the rate capacity at low current density is identical between the Li-3D CuO@Cu sponge anode and Li-planar Cu foil anode. However, at a high current density of 5 C, rate capacity of full cell with the Li-3D CuO@Cu sponge anode is as high as 80.3 mAh g^−1^, while that of the Cu foil holds only 54.8 mAh g^−1^ (Figure 5a). A remarkable 47% increase in specific capacity indicates the superiority of Li-3D CuO@Cu sponge anode. As shown in Figure 5b, the Li-3D CuO@Cu sponge anode realizes more stable cycling performance for full cells at 5C. After 120 cycles, the reversible capacity of the 3D current collector remains 87.8 mAh g^−1^, while the Li-planar Cu foil anode exhibits a sudden capacity attenuation after 95 cycles and the capacity drops to less than 40 mAh g^−1^. The sudden decay of Li-planar Cu foil's capacity is due to formation of dendritic Li. While Li dendrite in Li-3D CuO@Cu sponge anode is effectively inhibited, so the capacity remains high and stable. The galvanostatic charge and discharge profiles of the full cells at 5C are plotted in Figure S3 for the 5th, 50th and 100th. The 3D CuO@Cu sponge anode realizes not high but stable discharge and charge capacities (Zheng et al., 2019). At the same time, 3D CuO@Cu sponge anode has a lower voltage hysteresis in the charge/discharge process indicating the significant kinetic advantage of the Li_2_O-reinforced SEI (Song et al., 2018). The FESEM images of the Li-planar Cu foil anode and Li-3D CuO@Cu sponge before and after cycles are shown in Figures 5c–f. The original smooth surface of the Cu foil (Figure 5c) covered with mossy Li after 120 cycles (Figure 5d). For Li-3D CuO@Cu sponge anode, there is no difference between the morphologies before and after cycling (Figures 5e,f), indicates that the Li-3D CuO@Cu sponge anode has an efficacious SEI layer with sufficient mechanical strength that guarantees a homogeneous Li depositing process, therefore renders a superior cycle stability.

## Conclusions

In summary, we introduced a simple but effective strategy to suppress Li dendrite growth by using CuO@Cu submicron wire sponge as current collector. The 3D porous structure of the CuO@Cu submicron wire sponges, with SEI layer dominated by Li_2_O with strong mechanical strength, is conducive to homogenizing electric field distribution, therefore renders dendrite-free Li deposition. The Coulombic efficiency of the 3D porous CuO@Cu submicron wire sponge current collector remains 98% at current density of 2 mA cm^–2^, and 96% at current density of 5 mA cm^–2^ after 100 cycles. Even at an ultrahigh current density of 10 mA cm^–2^, the Coulombic efficiency still remains at 91% after 100 cycles. At a high current density of 5C, rate capacity of full cell with the Li-3D CuO@Cu sponge anode is as high as 80.3 mAh g^–1^, and after 120 cycles, the reversible capacity remains 87.8 mAh g^−1^. Compared with the planar Cu foils, CuO@Cu submicron wire sponge current collector displays superior electrochemical cycling performance with higher and more stable CE and longer service life. We believe this work can offer valuable guidance as well as deep understanding in design of novel materials or structures to suppress Li dendrite growth for further development of next-generation Li metal batteries, such as Li-S or Li-air batteries.

## Author contributions

CS developed the concept and designed the experiments. JY conducted the experiments. HY and JG built the cells and carried out the performance characterizations. YG and KX co-supervised the research. YG revised the work critically for important intellectual content. CS and HY co-wrote the manuscript. All authors discussed the results and commented on the manuscript.

### Conflict of interest statement

The authors declare that the research was conducted in the absence of any commercial or financial relationships that could be construed as a potential conflict of interest.
