# Supplementary material for: Li2O-Reinforced Solid Electrolyte Interphase on Three-Dimensional Sponges for Dendrite-Free Lithium Deposition
Source: Front Chem. 2018 Nov 6;6:517. doi: 10.3389/fchem.2018.00517 (PMC6233022; doi:10.3389/fchem.2018.00517)
Supplement: Supplementary file 1 [file Table_1.DOCX]

Supporting Information

Li_2_O‑Reinforced Solid Electrolyte Interphase on Three-Dimensional Sponges for Dendrite-Free Lithium Deposition

Chao Shen ^1*^, Huibo Yan ^1^, Jinlei Gu ^1^, Yuliang Gao ^1^, Jingjing Yang ^2^*, Keyu Xie ^1^

^1^ State Key Laboratory of Solidification Processing, Center for Nano Energy Materials, School of Materials Science and Engineering, Northwestern Polytechnical University and Shaanxi Joint Laboratory of Graphene (NPU), Xi’an, China.

^2^ School of Materials and Chemical Engineering, Xi’an Technological University, Xi’an 710032, China

*** Correspondence:**

Dr.Chao Shen, Dr. Jingjing Yang

[shenchao@nwpu.edu.cn](mailto:shenchao@nwpu.edu.cn), yangjingjing@xatu.edu.cn


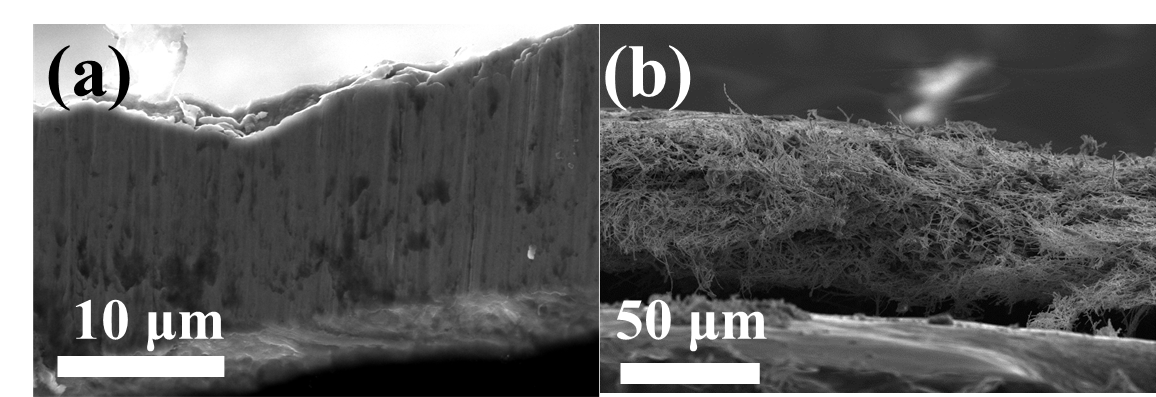


**Figure S1** The crossing-section of (a) planar Cu foil and (b) 3D CuO@Cu sponge. The thickness is about 15μm and 50μm, respectively.


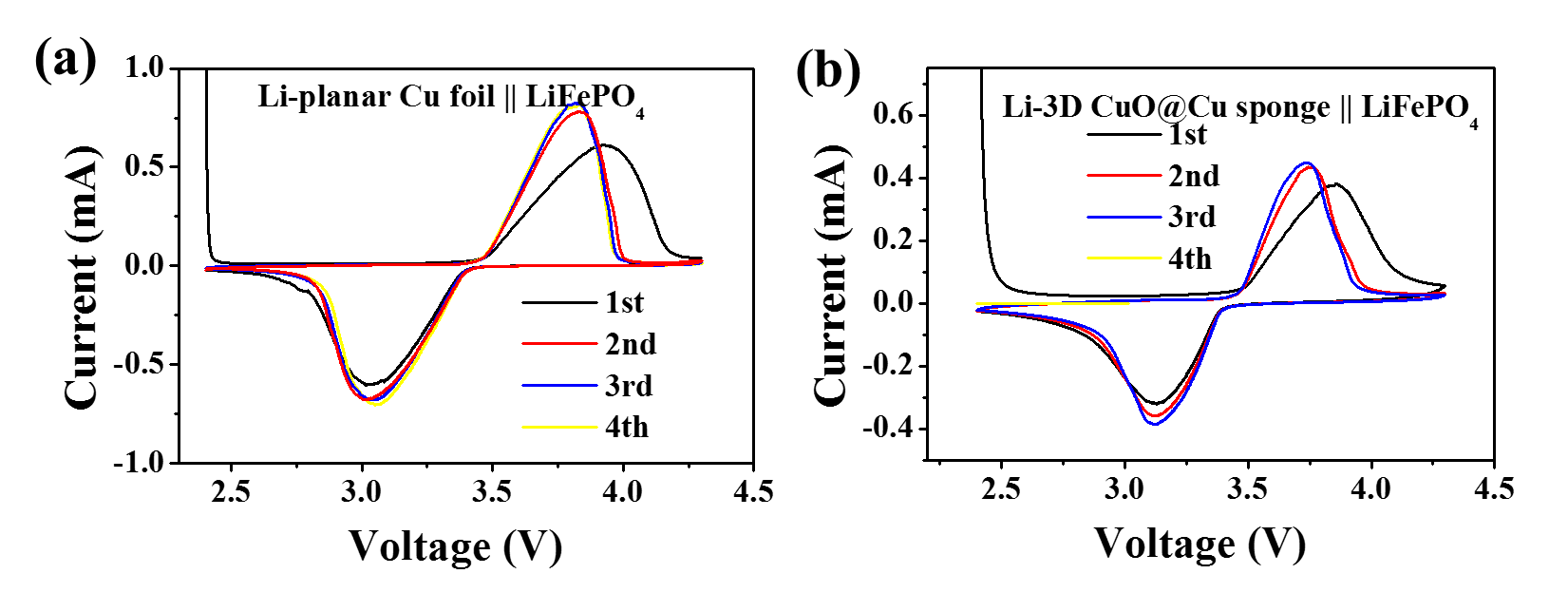


**Figure S2** The CV curves of Li-planar Cu foil || LiFePO_4_ and Li-3D CuO@Cu sponge || LiFePO_4_.


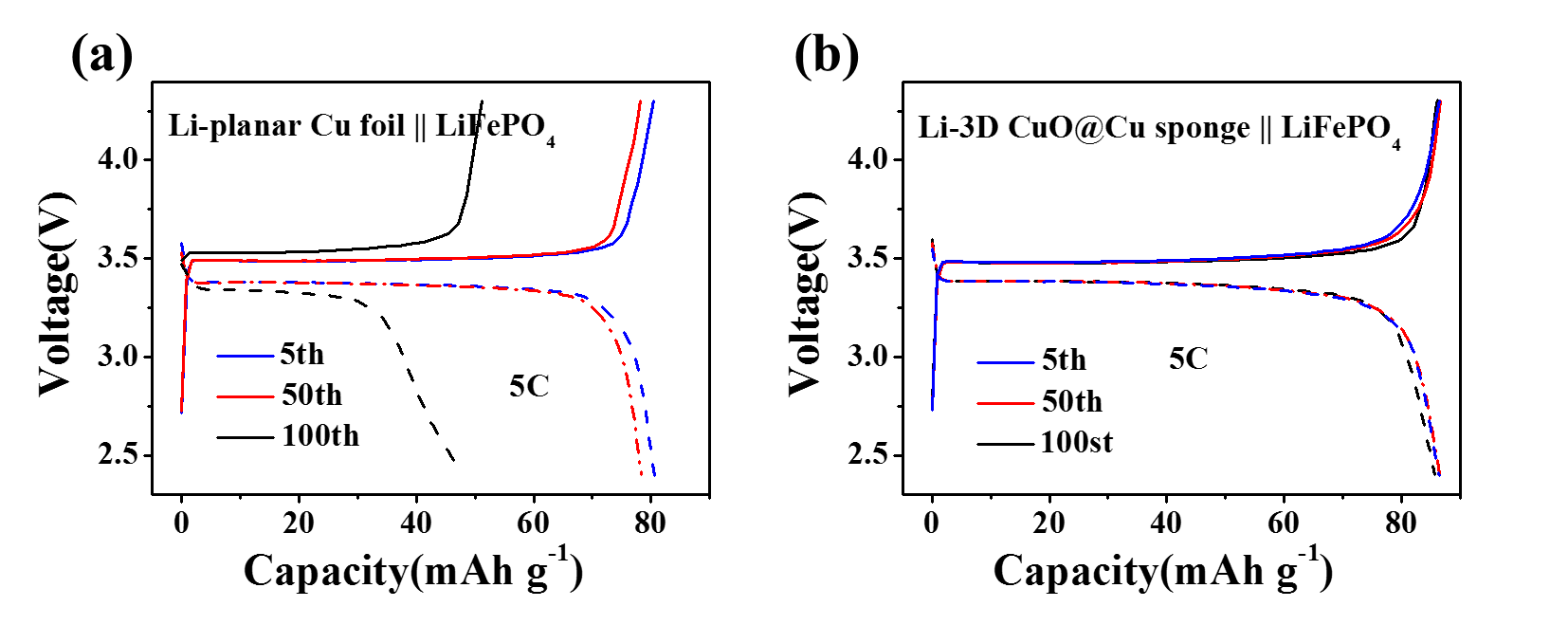


**Figure S3** The galvanostatic charge-discharge curves of Li-planar Cu foil || LiFePO_4_ and Li-3D CuO@Cu sponge || LiFePO_4_.
